# Age management practices toward workers aged 45 years or older: an integrative literature review

**DOI:** 10.47626/1679-4435-2020-536

**Published:** 2020-12-11

**Authors:** Danielli Rafaeli Candido Pedro, Nathalia Vasconcelos Fracasso, Raquel Gvozd Costa, Mariana Ângela Rossaneis, Patrícia Aroni, Maria do Carmo Fernandez Lourenço Haddad

**Affiliations:** 1 Programa de Pós-Graduação em Enfermagem, Universidade Estadual de Londrina (UEL) - Londrina (PR), Brasil; 2 Departamento de Enfermagem, Universidade Estadual de Londrina (UEL) - Londrina (PR), Brasil

**Keywords:** occupational health, aging, health management

## Abstract

Age management practices refer to the development and implementation of workplace strategies to support and improve the health and productivity of workers aged 45 years or older. The objective of this study was to analyze the scientific evidence available to support age management practices toward older workers. An integrative review was conducted, with the following databases searched in February 2019: LILACS, MEDLINE, Web of Science, and SCOPUS. Inclusion criteria consisted of original primary studies with full-text availability, published in Portuguese, English or Spanish. Secondary studies were excluded. No restrictions were imposed on publication dates given the paucity of literature on this topic. The final sample consisted of 11 primary studies published between 2006 and 2017, which addressed the following age management practices: workplace health promotion; employment exit and transition to retirement; knowledge transfer, training and lifelong learning; career development; flexible working time practices; and occupational safety and health management. Age management practices are promising tools to promote a work environment that is adequate to the needs of older workers.

## INTRODUCTION

The 21st century has seen a significant increase in population aging, with an estimated 962 million people worldwide aged 60 years or older.^1^ In Brazil, 13% of the population is over 60 years old, and by 2050, this figure is expected to reach 29.3%.^2^

The acceleration of population aging has also resulted in an aging of the overall workforce. Current estimates suggest that, starting in 2040, the majority of workers will be at least 45 years old, prompting a need for specific management practices to address the needs of these workers.^3^

As the workforce ages, strategies targeting disease prevention and health promotion in the workplace become increasingly necessary in order to preserve worker capacity and quality of life.^4^

In light of these observations, age will become an important factor in human resource management, demanding new policies, instruments and interventions to ensure a prolonged, healthy and productive working life.^5^

Organizations that wish to retain their employees for longer periods of time will have to adapt to their needs. These issues have given rise to the concept of age management, which refers to a set of practices to adapt environments to older workers and reduce age barriers in order to support and retain these professionals.^3^

The World Health Organization (WHO) defines older workers as individuals aged 45 years or older; according to the literature, this age marks the onset of physical and cognitive changes associated with aging.^4^^,^^6^

Benefits of age management for workers include increased motivation and satisfaction with work, improved work-life balance and the maintenance of work capacity and employability throughout one’s career. Age management also brings advantages to employers, including the ability to retain experienced workers and anticipate the scarcity of qualified professionals, reduce turnover and cut hiring and training costs, as well as recognize the strengths and weaknesses of different age groups in order to distribute tasks more effectively.^3^

To this end, the European Agency for Safety & Health at Work (EU-OSHA) published an electronic guide in 2016 to contribute to the implementation of age management practices, which consist of the following: recruitment of older workers; knowledge transfer, training and lifelong learning; career development; flexible working time practices; workplace health promotion; occupational safety and health management; job rotation and redeployment; and employment exit and transition to retirement.^7^

Age management strategies have become a crucial concern in the current context of workforce aging. Managers who know how to work effectively with different age groups in their companies will be able to achieve better results and improve the health and safety of employees, creating favorable conditions for the implementation of age management practices in Brazilian companies.

No studies to date have provided a picture of the current literature on age management, highlighting the need for an integrative review on the topic. The aim of this study was therefore to analyze the scientific evidence on age management practices in the literature.

## METHOD

This integrative review was conducted according to the following steps: description of the topic and development of the research question, collection of primary studies, data extraction and assessment, analysis of results and presentation of review findings.^8^

This review was guided by the following research question: “What scientific evidence is available to support age management practices?”

Inclusion criteria consisted of original primary studies which addressed the subject of the review, with full-text availability, and publication in Portuguese, English or Spanish. Secondary studies such as theses, dissertations, systematic, integrative or narrative reviews, letters to the editor and editorials were excluded. No restrictions were imposed on publication dates given the paucity of literature on this topic.

In February 2019, the following databases were searched: LILACS, MEDLINE, Web of Science and SCOPUS. The search was conducted using the controlled keywords “*saúde do trabalhador*” and “*envelhecimento*”, from the Health Sciences Descriptors (Descritores em Ciência da Saúde; DeCS) of the Virtual Health Library, and the MeSH terms “worker’s health” and “aging”. The uncontrolled keywords *saúde ocupacional*, occupational health, and age management were also used.

The LILACS database was searched using the following query: (*envelhecimento*) AND ((*saúde do trabalhador*) OR (*saúde ocupacional*)). No additional results were retrieved when the keyword “*gestão da idade*” was included in the query, and as such, it was excluded from the search parameters. The remaining databases were searched using the following query: (Worker’s health) AND ((Aging) OR (Age management)).

The primary studies retrieved in the search were independently screened by two experienced trained researchers, and a third rater was consulted to resolve any discrepancies. In the case of duplicate studies, only the version from the database with the largest number of publications was considered.

Articles were classified into evidence levels using the following system: level I (strongest) - systematic review or meta-analysis of randomized controlled trials or clinical practice guidelines based on systematic reviews of randomized controlled trials; level II - at least one well-designed randomized controlled trial; level III -well designed, non-randomized clinical trial; level IV - well-designed cohort and case-control study; level V - systematic review of descriptive and qualitative studies; level VI - a single descriptive or qualitative study; level VII - expert opinions and/or expert committee reports.^9^

Studies were analyzed descriptively and summarized in a table containing the following information: country, database, authors, year, objectives, main findings and age management practices analyzed. A summary of how the findings of each study relate to age management practices was also produced.

## RESULTS

This review included 11 primary studies, 4 of which were published in 2017, the year with the most publications^10^^-^^13^; this was followed by 2006 and 2015 with 2 publications^14^^-^^17^; and the years 2011, 2012 and 2016, with one article each.^18^^-^^20^

**Figure 1:**
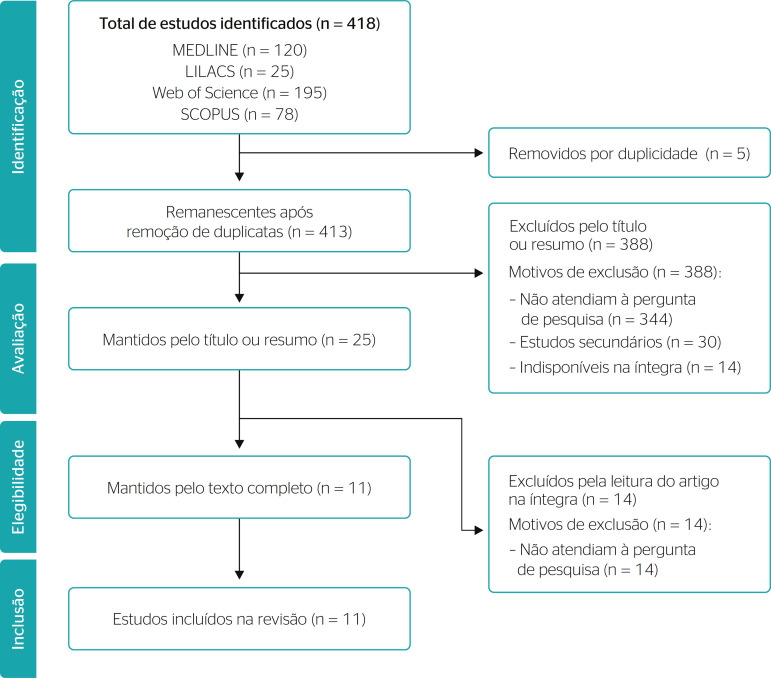
Flow diagram of the article selection process for this integrative review, as outlined by PRISMA (Preferred Reporting Items for Systematic Review and Meta-analyses) guidelines.

Figure 1 contains the flow diagram for this integrative review, presented according to PRISMA (Preferred Reporting Items for Systematic Review and Meta-analyses) guidelines.

Only 11 of the 418 articles retrieved in the search met inclusion criteria for the review. This speaks to the paucity of studies on age management in both the national and international literature.

The information extracted from the included studies and the age management practices addressed by each investigation are presented in Table 1.

**Table 1:** Summary of studies included in this integrative review of age management.

Country	Database	Reference	Objectives	Main findings	Age management practice
Finland	MEDLINE	Naumanen^15^	To describe the effects of health promotion for older workers.	Over 90% of participants reported that positive health habits and a good working environment play an important role in health promotion.	Workplace health promotion
Ireland	Web of Science	Mc Carthy^10^	To examine the association between job characteristics and mental health.	Multiple linear regressionanalysisshowed that job demands significantly predict symptoms of depression and anxiety.	Workplace health promotion
United States of America	Web of Science	Jinnett^11^	To analyze the combined effects of work safety, worker health and occupational demands.	Workplace safety and health conditions contributed to absenteeism and job performance.	Workplace health promotion; Occupational safety and health management,
South Korea	Web of Science	Park^20^	To recommend health strategies to support and improve the work capacity of aging employees.	The guidelines reinforce the importance of general medical examinations and healthy lifestyle promotion for workers.	Workplace health promotion
United Kingdom	Web of Science	Winkelmann-Gleed^18^	To examine the perspective of older workers on the factors that influence the decision to continue working or retire.	The participation of older workers in the labor market must be extended.Efforts must be made to create workplaces where employees feel valued and motivated to remain.	Workplace health promotion; Flexible working time practices; employment exit and transition to retirement.
Netherlands	SCOPUS	Ybema^12^	To investigate the efficacy of human resource practices in improving sustainable employment.	Twenty-three practices were studied. Improvement rates ranged from 25% for nutritional support to 89% for providing a good internal working environment.	Workplace health promotion; Knowledge transfer, lifelong training; career development.
China	SCOPUS	Peng^13^	To analyze age differences in the association between emotional suppression, work-related physical stress and affective well- being.	Emotion suppression was negatively associated with physical stress in older workers. In sample 1, the frequency of emotional suppression was positively associated with well- being in older workers.	Workplace health promotion
Australia	SCOPUS	Baxter^16^	To develop a calculator to assist with investments in workplace health.	The calculator can estimate the potential annual savings resulting from the implementation of a health promotion program.	Workplace health promotion.
Australia	SCOPUS	Van Holland^19^	To examine the efficacy and cost-benefit ratio of the POSE (Promotion of Sustained Employability) program	The program consists of a comprehensive assessment process followed by a counseling session and, if necessary, a personalized intervention.	Workplace health promotion.
Finland	SCOPUS	Naumanen^14^	To define and describe the effects of health promotion initiatives on older workers.	Worker health can be improved through lifestyle habits, positive attitudes, personal relationships and education.	Workplace health promotion.
Brazil	LILACS	Gvozd^17^	To examine how workers feel toward retirement.	The results revealed feelings such as freedom, satisfaction, anxiety, fear and unhappiness.	Employment exit and transition to retirement.

Ten of the articles were published in English^10^^-^^16^^,^^18^^-^^20^ and only one study was published in Portuguese and conducted in Brazil,^17^ reflecting the paucity of the national literature on age management.

The analysis of evidence levels revealed that 9 studies provided level VI^10^^-^^18^; evidence, one provided level VII evidence in the form of expert recommendations^20^; and one randomized clinical trial provided level II evidence^19^ of age management practices.

The dimensions of age management addressed by the articles included workplace health promotion, which was discussed in 10 studies^10^^-^^18^^,^^20^; employment exit and transition to retirement, analyzed by 2 studies^17^^,^^18^; and knowledge transfer, lifelong training, career development,^12^ flexible working time practices,^18^ occupational safety and health management,^11^ examined by one study each. The recruitment of older workers, job rotation and redeployment were not addressed by any of the studies included in this review.

## DISCUSSION

The changes proposed in the recent reform of the Brazilian pension system include an extension of contribution periods, with workers having to stay active for longer in order to retire. These individuals will therefore need to remain in the workforce for longer periods of time.^21^

These circumstances highlight the need to identify management practices and strategies that contribute to worker retention and help workers remain active and productive in their respective jobs. Therefore, the aim of this study was to identify existing initiatives in this area, with a focus on age management practices and their benefits.

To this end, included studies will be discussed according to the specific age management practices they chose to address. The practices examined in the studies reviewed included the following: workplace health promotion; occupational safety and health management; flexible working time practices; knowledge transfer, training and lifelong learning; career development; employment exit and transition to retirement; recruitment of older workers; and job rotation and redeployment.

The first dimension of age management identified in the studies reviewed was workplace health promotion. The literature on the subject showed that the work environment is an important setting for measures that improve worker quality of life, health and safety. These measures include promoting the approximation of workers and health care services by monitoring clinical exams and raising awareness of the importance of health care, nutrition, physical activity and disease prevention.^11^^,^^14^^-^^16^^,^^19^^,^^20^

Another important initiative is the implementation of health promotion programs that provide continued psychological support for workers, promoting emotional strengthening and reducing the negative impact of job demands and work-related stress.^10^^,^^12^^,^^13^^,^^18^ These results corroborate those of a study involving psychologists in the Brazilian state of Ceará, who observed that psychological services contributed to worker health promotion, and recommended that companies implement worker health initiatives in a permanent, regular and systematic manner.^22^

The introduction or expansion of institutional programs to improve worker health can reduce health care costs, occupational accidents and absenteeism due to health issues.^23^ The success of workplace health promotion programs depends on the workers’ commitment to changing or improving their habits, but also on the support of managers who help create an organizational culture that supports worker well-being.^24^

The second component of age management addressed in the studies reviewed was occupational safety and health management. An important strategy in this regard was the constant assessment of the occupational risks to which workers are exposed, followed by adaptations or adjustments to the work environment in order to improve safety and health conditions.^7^

Studies which examined these issues found that inadequate safety conditions contributed to absenteeism and reduced performance, while safe workplaces that considered the skills and workloads of employees and offered health promotion programs had lower absenteeism and higher productivity rates. The studies also emphasized the need to eliminate accident causes in order to create a healthy working environment.^11^ These observations corroborate the conclusions of a review on the role of nurses in risk prevention, which found that these professionals make a crucial contribution to the prevention, reduction and elimination of ergonomic risks, highlighting their importance in organizational settings.^25^

The first step in ensuring a safe working environment is the assessment of the risks to which workers are exposed, while considering the diversity of workers and the needs of every age group. Risk assessment allows for the implementation of changes such as replacing old equipment, equipping the work environment for employees of all ages, automating repetitive tasks and ensuring adequate lighting.^7^

Work safety practices should aim to reduce occupational accidents and illnesses, while also preserving the mental health of workers. These measures should also consider the skills of workers in order to help them carry out their activities safely and efficiently.^26^ The small number of work safety studies identified in this review highlights the need for further research on how employers can improve the health and safety of working conditions for older employees.

The third category, flexible working time practices, was discussed in a study which identified it as a potential solution for the management of older workers. However, this is still a remote possibility, since few institutions have the necessary conditions to implement it. The study mentions that flexible hours could allow older workers to stay home for longer periods of time, contributing to the adaptation and transition to retirement, an important component of age management with which flexible working time practices are clearly associated.^18^

A study that compared the implementation of flexible working time practices across multiple European countries demonstrated that the autonomy in determining working hours could improve the work-life balance for employees.^27^ Flexible working time practices include giving workers the freedom to choose when they start and end work on a given day, as well as where they will carry out their activities, while keeping the same total daily hours. These agreements are negotiated between employers and employees, considering the applicability of these practices to the particular workplace. Flexible working hours can increase worker commitment and awareness of their role in the institution.^28^ The work hours demanded by an institution may conflict with workers’ needs for family and social interaction; however, flexible working time practices can help balance these needs, becoming an important human resource management tool.^29^

The fourth and fifth components of age management addressed by the studies reviewed were knowledge transfer and lifelong learning, as well as career development. These practices were combined into a single category since each was only addressed by one study.

As companies strive to retain qualified professionals who are also attuned to technological developments in the modernization of work processes, the promotion of continuous training has emerged as an important management strategy to ensure that older workers continue to play an active and participative role in the work force. In addition to institutional initiatives, it is important that workers themselves commit to investing in continuous training.^30^ Training programs offered by institutions should be in line with the needs of older workers, encouraging the acquisition of relevant knowledge in order to promote the career growth and development.^31^

The sixth category, employment exit and transition to retirement, was addressed by two studies which focused on the preparation of older workers for retirement and work exit. The pre-retirement period should be dedicated to the planning of life in retirement and to deciding on the best time to leave one’s job.^7^^,^^32^

Brazilian researchers conducted a study of workers in a public university to explore their feelings and perspectives toward retirement, finding that most participants expressed positive feelings such as freedom and satisfaction. It was also noted that workers can exercise agency in their career decisions by keeping their jobs even after retiring. Another relevant finding was that most pre-retirement workers reported no preparations for retirement. The few who did prepare were primarily concerned with seeking information about financial and emotional issues. These data underscore the need for the implementation of retirement preparation programs in companies.^17^

Another study, which aimed to present the perspective of older workers in the United Kingdom about the influence of work commitments on the decision to retire, reported that financial insecurity was the most influential factor in this decision, and that workers adopt a progressive approach to retirement out of fear. The transition to retirement may also be negatively impacted by feelings of commitment to the organization, to colleagues and their profession, described by the authors as feelings of moral responsibility for the institution.^18^

These findings agree with those obtained in a study conducted in South Korea, Japan, Germany, the United States and United Kingdom, which found that workers do prepare for retirement, but do not usually have an effective retirement plan. It is therefore essential that policies and educational measures to provide support in the preparation for retirement are made available to workers.^32^

The increased free time experienced by workers after retirement can also have a negative impact on individual health. This further highlights the need for retirement planning programs to help individuals understand the physical and mental health issues they may experience after employment exit, addressing issues such as post-career activities and financial education, in order to reduce the negative effects of retirement.^33^

Despite the growth in the aging -and retired -population, few Brazilian companies have implemented retirement preparation programs, demonstrating the need for further scientific research to support the introduction of these practices in Brazilian companies.^34^

The seventh component refers to the recruitment of older workers. Currently, the hiring process in most companies is predominantly skewed toward younger workers, since some managers still believe that older workers are associated with decreased productivity. Yet older workers can bring several advantages to employers, such as the ability to rely on a wealth of accumulated experience and in-depth knowledge of work processes, a commitment to ethical principles, low absenteeism rates, and increased loyalty and reliability due to the corporate maturity and emotional strength acquired throughout their careers.^35^ Despite the relevance of the issue of hiring older workers, this topic was not addressed in any of the articles selected.

The eighth and last dimension of age management, job rotation and redeployment, pertains to the transfer of workers between shifts or areas in the organization in a planned manner at regular intervals, based on a pre-established arrangement between employer and employee, so as to increase productivity and improve performance. Though this can be advantageous to both workers and employers, its implementation in organizations has not been sufficiently examined by scientific research.^7^

All but one of the studies selected (n = 11) were conducted outside of Brazil, suggesting that developed countries may be more interested in the study of workforce aging, and consequently, are actively seeking to extend the working life of employees, improve their quality of life, and increase the productivity and participation of older adults in the workforce. In fact, these have been important goals in European policy since the late 1990s, which marked the beginning of the workforce aging process.^7^

Limitations of this study include the scarcity of studies with high levels of evidence, and the predominance of descriptive studies with little statistical sophistication. There is a need for additional studies to affirm and extend current knowledge on age management practices and their implementation in organizations, with a special focus on the recruitment of older workers, job transfer and redeployment. The absence of studies into these practices, as identified in this review, points to a gap in the literature on age management practices. We also found that a significant number of articles (n = 14) were not available in full text, which can be a limitation, since these studies could have made important contributions to this review.

Nevertheless, this study makes a relevant contribution to the literature by synthesizing primary articles that discuss age management practices that can adjust and improve working conditions for older employees, compiling scientific evidence to support the implementation of these strategies.

## CONCLUSION

The age management practices discussed by articles in this review included workplace health promotion, occupational safety and health management, flexible working time practices, knowledge transfer, training and lifelong learning, career development, employment exit and transition to retirement. These practices have emerged as promising management strategies to ensure that workplaces are adequate to the needs of older workers, which could ultimately benefit all age groups, improving quality of life and promoting continued career growth.

Age management practices can also help reduce occupational accidents and illnesses, cut recruitment and training costs, and improve the retention of experienced and qualified professionals who are motivated to stay active and productive. We also conclude that the scarcity of age management studies in Brazil reflects a lack of concern with the study of these practices and their implementation, and a low interest in investing in these issues, on the part of local researchers and organizations.
